# Implications of Temperature and Prey Density on Predatory Mite *Amblyseius swirskii* (Acari: Phytoseiidae) Functional Responses

**DOI:** 10.3390/insects15060444

**Published:** 2024-06-12

**Authors:** Mohammed M. E. Elmoghazy, Dalia Mahmoud Abdelmonem Elsherbini, Abadi M. Mashlawi, Ateya Megahed Ibrahim, Ahmed A. El-Mansi, Mohamed El-Sherbiny

**Affiliations:** 1 Agriculture Zoology and Nematology Department, Faculty of Agriculture, Al-Azhar University, Cairo P.O. Box 11884, Egypt; mmeelmoghazy@gmail.com; 2 Department of Clinical Laboratory Sciences, College of Applied Medical Sciences, Jouf University, P.O. Box 2014, Sakaka 72388, Saudi Arabia; 3 Department of Biology, College of Science, Jazan University, Jazan 45142, Saudi Arabia; amashlawi@jazanu.edu.sa; 4 College of Nursing, Prince Sattam bin Abdulaziz University, Al-Kharj 11942, Saudi Arabia; a.eleglany@psau.edu.sa; 5 Department of Family and Community Health Nursing, Faculty of Nursing, Port Said University, Port Said 42511, Egypt; 6 Biology Department, Faculty of Science, King Khalid University, Abha 61413, Saudi Arabia; aelmansi@kku.edu.sa; 7 Department of Basic Medical Sciences, College of Medicine, AlMaarefa University, P.O. Box 71666, Riyadh 11597, Saudi Arabia; msharbini@um.edu.sa; 8 Department of Anatomy, Faculty of Medicine, Mansoura University, Mansoura 35516, Egypt

**Keywords:** *Amblyseius swirskii*, *Tetranychus urticae*, functional response, temperature, mites

## Abstract

**Simple Summary:**

*Amblyseius swirskii* Athias-Henriot (Acari: Phytoseiidae) is one of the most potent predaceous mites in biological pest management, feeding on phytophagous mites, pollens, and plant exudates. *Tetranychus urticae* Koch (Acari: Tetranychidae), a global mite, infects many field crops, fruit orchards, and ornamental plants by reducing photosynthesis and feeding on plant cells. This study reveals that the functional response estimates of *A. swirskii* vary with temperature (14, 18, 22, 26, 30, and 34 ± 1 °C) and prey density. We found that the highest searching efficiency was at 26 °C and that the lowest was at 14 °C. The handling time for each prey item also varies with temperature and prey density. The functional response curves align with the type II functional response model, showing the inverse dependence of temperature and prey density. The predation curves for *A. swirskii* show a significant difference in the mean numbers of *T. urticae* consumed at different prey densities and temperatures. This study highlights the relationship between *A. swirskii* and *T. urticae* and the importance of temperature and prey density in natural enemies’ activities. The findings can help predict *A. swirskii* behavior and its effectiveness in controlling *T. urticae* populations.

**Abstract:**

*Amblyseius swirskii* are predaceous mites that feed on phytophagous mites, pollens, and plant exudates and are known as one of the most potent biological pest management agents. *Tetranychus urticae* is a global mite that is difficult to manage because of its high population growth rates, necessitating alternative management measures like biological control. Regarding the functional response, the effects of temperature and prey density are some of the essential behaviors of natural enemies. This study investigates the effect of varying temperatures and prey densities on *A. swirskii*, a biological control agent for *T. urticae*. The present results demonstrated the change in the functional response estimates when *A. swirskii* was reared at various temperatures and different prey densities. The results of the estimates regarding the searching efficiency (*a′*) showed the highest value (*a′* = 0.919) at 26 °C and the lowest value (*a′* = 0.751) at 14 °C. The handling time per prey item (*T_h_*) for the predatory mites changed with the temperature and prey density, showing the shortest handling time at 26 °C (*T_h_* = 0.005) and the highest value at 14 °C (*T_h_* = 0.015). The functional response curves matched the type II functional response model, demonstrating the inverse dependence of temperatures and prey density with a positive quadratic coefficient. The predation curves for *A. swirskii* showed a significant difference between the mean numbers of *T. urticae* consumed at various prey densities and temperatures, illustrating a relationship between *A. swirskii* and *T. urticae.* Therefore, the results of this research may be utilized to forecast the behavior of *A. swirskii* and its usefulness in controlling *T. urticae* populations.

## 1. Introduction

*Tetranychus urticae* is a globally prevalent mite of the polyphagous species of the family Tetranychidae that may severely harm the leaves, buds, and fruits of numerous horticultural crops grown in open fields and greenhouses [1,2,3]. It feeds on around 1100 host plant species, including over 150 economically important ones [4]. Managing a decrease in crop photosynthetic features by nurturing plant cells is challenging due to their elevated population rates of growth due to various cultivars’ haplodiploid sex determination mechanism, their short life cycle, and the rapid emergence of pesticide resistance [5,6,7], necessitating the employment of alternative management measures like biological control [8].

One of the predaceous mites feeding on tiny insects and phytophagous mites belonging to the Phytoseiidae family is widely distributed worldwide, with about ninety genera, including 2730 species [9]. Most of these family members are crucial for spider mite biological control in greenhouse crop production, and certain species also feed on microsoil inhabitants, pollens, and plant exudates [10,11].

Several factors, including temperature [12], humidity [13], and variable plant texture [14], influence the functional response of phytoseiids. Sentis et al. [15] used the functional response to investigate the impact of temperature on predation traits. Temperature is an important element of metabolic action in the ecological metabolic theory, as it helps determine the predator’s energetic efficiency [16]. Few studies have revealed a significant correlation between temperature and the functional response of phytoseiids [12,17,18].

After being marketed in 2005, *Amblyseius swirskii* was proven to have the most effective biological control in protected agriculture [19], with its excellent predatory mite and prey consumption capabilities. It can reproduce and feed on *Tetranychus urticae*, insects, pollen, plant exudates, and honeydew [10,20]. Even when high-quality alternative food such as pollen is present, it targets all prey mites [21]. It exhibits the highest fecundity, pre-adult survival rate, and predation capacity toward adult *T. urticae* compared to three species from the genus *Neoseiulus* [22], and it has recently been extensively utilized as an augmentative biological control agent [23].

*Amblyseius swirskii* completes its development from egg to adult under varying temperatures from 15 to 37.5 °C, with the highest survival rate and the shortest development period recorded at 25 to 30 °C, according to the difference in nutrition [24,25,26].

The functional response emphasizes the importance of the link between the individual’s consumption rate and food density [27,28]. In interactions between predator and prey, the functional response offers information on a natural enemy’s biological control efficacy against a specific pest [29]. Furthermore, it can indicate predators’ search efficiency and predation rates, and assessing predator behavior is a vital initial stage in identifying predators’ ability to control prey [30]. Previous researchers demonstrated the functional response of *A. swirskii* feeding on the tetranychids *Tetranychus turkestani* [31], *Eotetranychus frosti* [32], astigmatid *Suidasia medanensis* [33], and eriophyid *Aculops lycopersici* [34].

The key purpose of this study was to determine the effect of varying the temperature and prey density of *Amblyseius swirskii*, as a biological control agent of *Tetranychus urticae*. Regarding the functional response, the effects of temperature and prey density are some of the significant activities of natural enemies. Since previous studies were carried out at constant temperatures, this study tested varying temperatures to forecast the behavior of *A. swirskii* and its usefulness in controlling *T. urticae* populations. Analyzing functional and numerical responses provides information on the prey–predator relationship, which is necessary to apply a biological control agent effectively.

## 2. Materials and Methods

### 2.1. Laboratory Rearing of Prey and Predator Mites

Mite cultures were developed in the laboratory at a temperature of 23 ± 2 °C, 60 ± 5% RH, and a photoperiod of 16:8 h (light: dark) for 24 h using *T. urticae* and *A. swirskii* collected from field plants. *T. urticae* was reared on bean plants grown in the laboratory (*Phaseolus vulgaris* L.) for three weeks before being used as food. Eighteen to twenty of these potted plants were provided with mixed stages of *T. urticae.*

The predatory mite *A. swirskii* was reared on detached mulberry leaves. Various fresh mulberry leaves were washed with a water spray to clean them before use and then left to dry. Each leaf was placed on a layer of regularly moistened cotton wool in foam dishes (20 × 15 cm in diameter and 2 cm in depth), as moisture keeps the leaves fresh for about a week and stops mite escape. Mixed stages of *T. urticae* on kidney bean leaves from the rearing colony were placed in each dish to rear predator mites at all the developmental stages as needed. At least 8 to 10 generations of *A. swirskii* were generated in this way before using the colony in this experiment.

### 2.2. Functional Response Experiment

The bean plant leaf discs (4 cm diameter) on a wet cotton wool layer in Petri dishes (10 cm diameter—2 cm depth) were placed in controlled-temperature incubators at 14, 18, 22, 26, 30, and 34 ± 1 °C, 60 ± 5% RH, and with a photoperiod of 16:8 h (light: dark) for 24 h [14,35] to measure the effectiveness of the predator with the temperature change. With eight newly emerging densities of *T. urticae* individuals able to determine the functional response of *A. swirskii*, experiments were conducted (5, 15, 25, 35, 45, 55, 65, and 75) at immature stages, including at the larvae, protonymph, and deutonymph stage, at about similar proportions.

Specifically, density 5 included 1, 2, 2; density 15 included 5, 5, 5; density 25 included 8, 8, 9; density 35 included 11, 12, 12; density 45 included 15, 15, 15; density 55 included 18, 18, 19; density 65 included 21, 22, 22; and density 75 included 25, 25, 25 larvae, protonymphs, and deutonymphs, respectively. Based on our observations, this method was employed because *A. swirskii* favored the protonymph stage of *T. urticae* over the other developmental stages.

Prey mites were placed onto plant leaf discs with a delicate soft brush. A single newly emerged adult female, *A. swirskii*, starved for 24 h, was released into each plant disc. After 24 h, the *A. swirskii* were removed from the plant leaf discs, and the number of eaten larvae and nymphs was counted; all immature stages were combined in the final counts. The analysis did not include plant leaf discs from which a live *A. swirskii* was not recovered because of loss or death. Each prey density was replicated ten times.

### 2.3. Data Analysis

The *A. swirskii* functional response to prey densities and different temperatures was calculated using Holling’s equation [36].

$$P_{e}=\frac{a^{\prime }\;N{\;T}_{tot}}{1+a^{\prime }{\;T}_{h}\;N}$$

$P_{e}$ = number of prey eaten during a searching period.

$a^{\prime }$ = attack rate or searching efficiency.

N = density of prey.

$T_{tot}$ = total time spent.

$T_{h}$ = handling time per prey item.

Statistical analysis was conducted using the SPSS program (version 25). A one-way ANOVA test followed by a post hoc–least significant differences LSD test was performed to compare the statistical differences between groups and an independent sample *t*-test was performed. *p*-value ≤ 0.05 was considered statistically significant. GraphPad Prism 8.0.2(263) and Microsoft Excel programs were used for making graphs.

## 3. Results

The influence of the *Tetranychus urticae* density on the *Amblyseius swirskii* feeding capabilities was studied using six different temperatures (Table 1). One-way ANOVA revealed significant differences between the groups of densities (5, 15, 25, 35, 45, 55, 65, and 75), as seen by the (F) values of 1245.19, 1732.94, 2205.66, 5359.50, 4052.85, and 2912.52 (*p* < 0.001) at 14, 18, 22, 26, 30, and 34 °C, respectively.

The eating ability of *A. swirskii* females when fed on *T. urticae* immature stages was highly influenced by temperature changes. The prey consumption rate reached a maximum of 26 °C on almost all densities. Then, it decreased as the temperature increased, as shown in Table 1.

**Figure 1:**
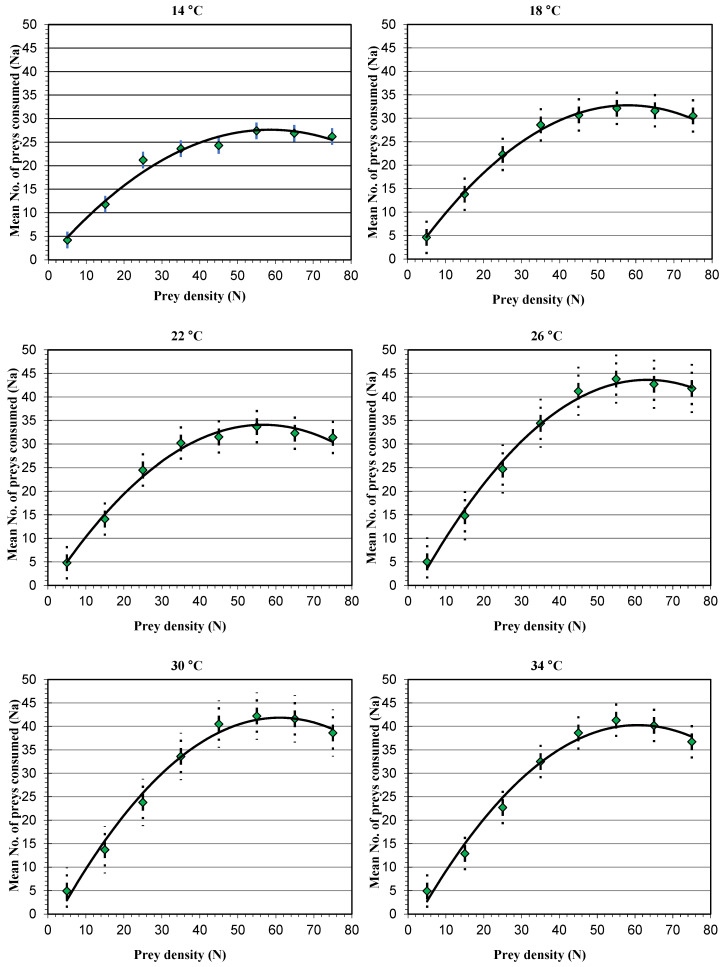
The functional response model of *A. swirskii* for the difference in temperatures and the numerical density of *T. urticae* matched the type II functional response model according to Holling’s model’s linearization. Error bars show ± S.E.M of the combined loss of individuals for each prey density.

**Figure 2:**
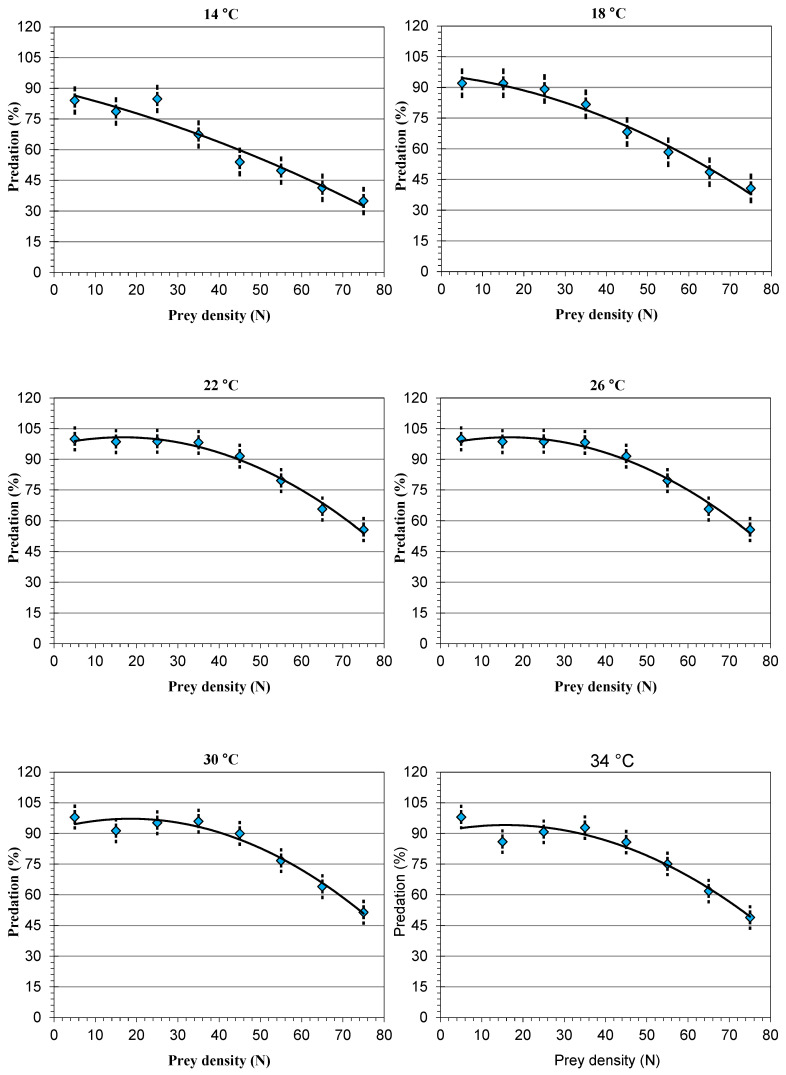
Linearization models for the density of *T. urticae* and the percentages (%) of predation by *A. swirskii* with the temperature difference. Error bars show ± S.E.M of the combined loss of predation percentage for each prey density.

The linearization link between the difference in temperatures and the prey’s densities regarding the predator’s functional response fitted the type II functional response model, demonstrating the inverse dependence of temperature and prey density, in addition to a positive quadratic coefficient (Table 2 and Figure 1 and Figure 2). As revealed by the functional response curves, *A. swirskii* responded more strongly at lower prey densities.

**Figure 3:**
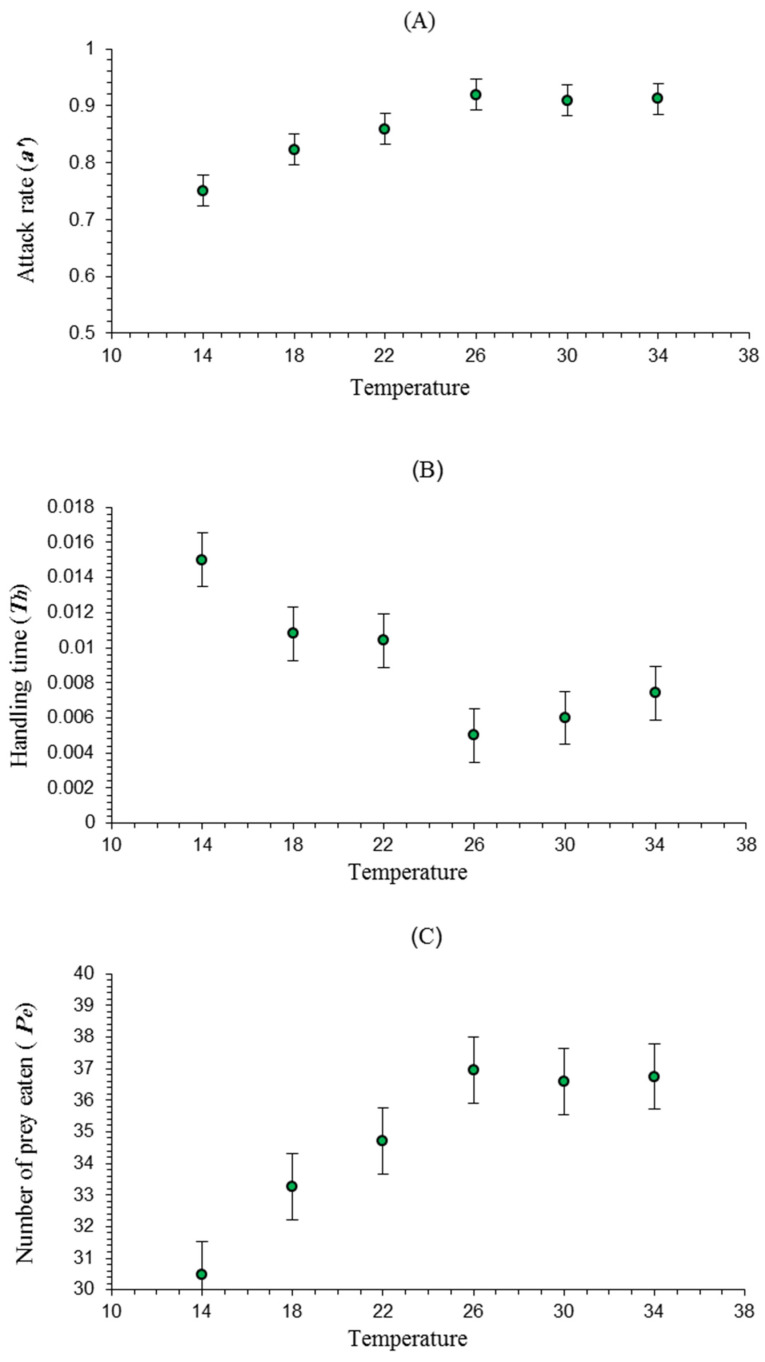
(**A**–**C**) Shows the parameters of the equation for *A. swirskii*: attack rate (*a*′), handling time (*T_h_*), and number of *T. urticae* eaten during a period of searching (*P_e_*), respectively. The points indicate coefficients with standard errors based on functional response models that characterize the link between temperatures and the functional response coefficients.

The functional response parameter estimates of *A. swirskii* changed with a varying temperature (Table 2 and Figure 3). The handling time/prey item at 26 °C was the shortest (*T_h_* = 0.005), followed by *T_h_* = 0.006 at 30 °C, *T_h_* = 0.007 at 34 °C, *T_h_* = 0.010 at 22 °C, *T_h_* = 0.011 at 18 °C, and *T_h_* = 0.015 at 14 °C. The maximum searching efficiency (*a′*) was recorded at 26 °C (*a′* = 0.919), followed by 34 °C (*a′* = 0.912), 30 °C (*a′* = 0.910), 22 °C (*a′* = 0.859), and 18 °C (*a′* = 0.823), with the lowest search rate at 14 °C (*a′* = 0.751). This model showed a strong match to the data, as seen by the high (*R^2^*) values of 0.996, 0.993, 0.990, 0.989, 0.986, and 0.976 for 18, 26, 22, 30, 34, and 14 °C, respectively.

A comparison of the functional response curves of *A. swirskii* showed a significant difference at different temperatures (Figure 1). The following were the results of the Independent Samples T Test comparing between temperatures: at temperatures of 14 °C and 18, 22, 26, 30, and 34 °C, the results were (F = 4.67; df = 152.58; *p* ≤ 0.05), (F = 4.78; df = 150.64; *p* ≤ 0.05), (F = 42.61; df = 125.34; *p* < 0.001), (F = 39.44; df = 127.82; *p* < 0.001), and (F = 35.94; df = 130.48; *p* < 0.001), respectively. Also, at temperatures of 18 °C and 22, 26, 30, and 34 °C, the results were (F = 0.015; df = 157.82; *p* = 0.902), (F = 20.23; df = 140.21; *p* < 0.001), (F = 17.67; df = 142.70; *p* < 0.001), and (F = 14.99; df = 145.24; *p* < 0.001), respectively. At temperatures of 22 °C and 26, 30, and 34 °C, the results were (F = 17.98; df = 142.80; *p* < 0.001), (F = 15.57; df = 145.21; *p* < 0.001), and (F = 13.09; df = 147.62; < 0.001), respectively. At temperatures of 26 °C, 30, and 34 °C, the results were (F = 0.133; df = 157.83; *p* = 0.716), and (F = 0.572; df = 157.30; *p* = 0.451). Finally, at temperatures of 30 and 34 °C, the result was (F = 0.154; df = 157.82.; *p* = 0.695).

**Figure 4:**
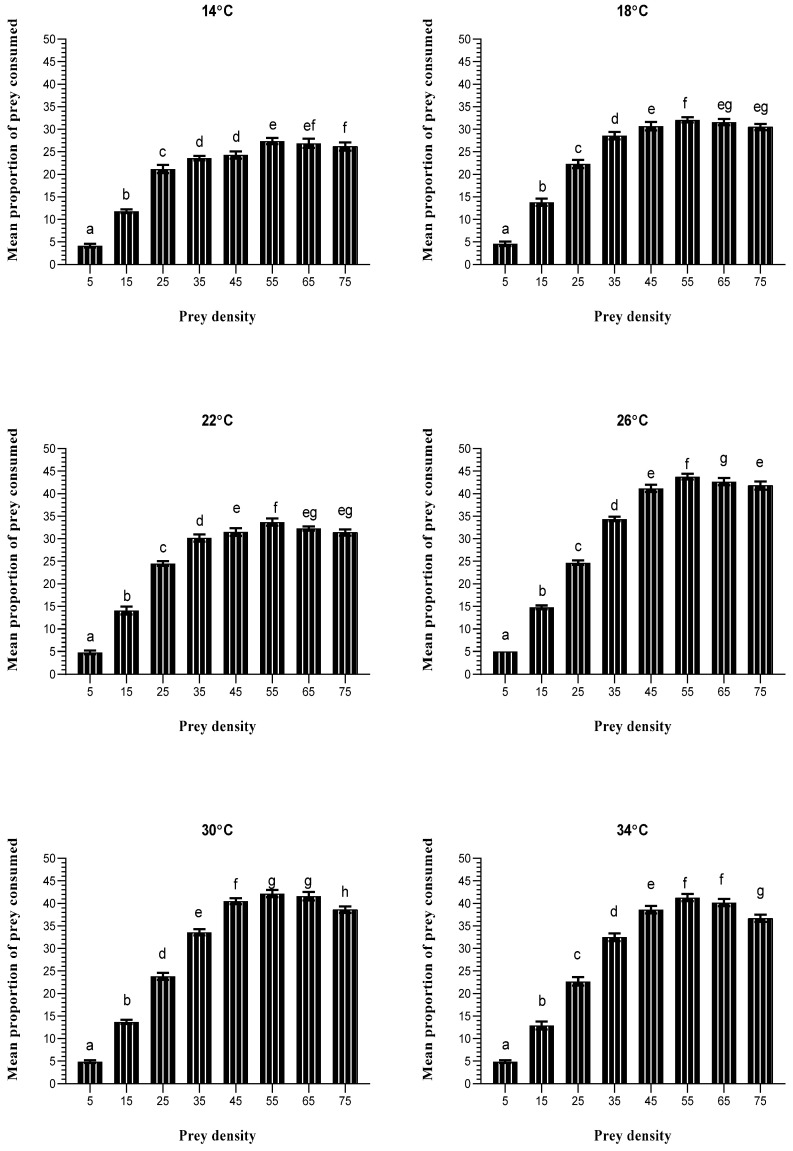
Mean ± SD. No. of *T. urticae* consumed by *A. swirskii* at various temperatures and diverse prey densities. According to the one-way ANOVA and the LSD post hoc test, different letters in the same row mean a significant difference (*p* ≤ 0.05).

Figure 4 demonstrates the influence of the density of immature *T. urticae* stages on the number of prey consumed by *A. swirskii* at six different temperatures. Upon comparing the average number of prey killed at each temperature in the columns, the data revealed differences in the predator’s functional responses across all temperatures. There was a significant difference at 14 °C in the number of prey killed between densities (5, 15, 25, 35, 55, and 75; *p* < 0.001), at 18 and 22 °C between densities (5, 15, 25, 35, 55, and 65; *p* < 0.001), at 26 °C between densities (5, 15, 25, 35, 55, 65, and 75; *p* < 0.001), and at 30 and 34 °C between densities (5, 15, 25, 35, 45, 55, and 75; *p* < 0.001). As a result, the density of *T. urticae* at the same temperature affects the functional response of *A. swirskii*.

## 4. Discussion

This study demonstrated that the functional response of *Amblyseius swirskii* matched the type II functional response, demonstrating that varying temperatures alter the type of functional response. Differences in the predator’s functional reaction at all prey densities were also seen.

A prior study showed that *A. swirskii* exhibits a type II functional response when exposed to varying densities of *T. urticae* [20,37]. Xiang et al. [22] found that *A. swirskii* had a relatively higher fitness and better predatory abilities toward *T. urticae* compared to other Phytoseiidae. The functional response of the second type was recorded in numerous species of phytoseiid mites, as in the females of *N. barkeri* that were released on various densities of *T. urticae* at different developmental stages under laboratory conditions on leaf discs of pepper plant at different temperatures (20, 25, 30, and 35 °C), according to Faraji et al. [12]. Furthermore, *N. cucumeris* feeds on *T. tabaci*, while *N. cucumeris*, *N. barkeri*, and *E. nicholsi* feed on *T. flavidulus* [38,39]. According to several studies, the type of functional response and the precise parametric values of a predator may differ with numerous determinants, such as the experimental environment and specific temperatures [40,41], host plant on which the prey and predator interact [14,42], and predator generation [43].

In this study, the feeding of *A. swirskii* on the immature stages of *T. urticae* was significantly affected by differences in temperature. It was clear that the rate of prey consumption increases with increasing density up to a certain threshold, after which it is insignificant. It then decreases with an increasing population density of *T. urticae* at 14, 30, and 34 °C. At 18, 22, and 26 °C, the predation rate began to decline significantly immediately after it exceeded a certain threshold. This may be due to the interference in its ability to prey, thus increasing the satiety of the predator.

A previous study conducted by Mumtaz et al. [44] on Phytoseiidae (*Neoseiulus californicus*) reported that the predator exerts significant control on prey populations at low *T. urticae* densities (4, 5, 8, and 10) because the predator can effectively control and reduce the number of prey when the prey density is low. However, as the prey density increases beyond a certain point, the predator’s predation rate increases, reducing its efficiency in controlling the prey.

These results are consistent with previous studies, which showed that temperature influences predator–prey consumption [18,45,46]. They found that when temperatures were raised from 15 to 25 °C, the daily consumption rates of *T. urticae* eggs and nymphs by the predators *Phytoseiulus persimilis*, *Phytoseius plumifer*, and *Typhlodromus bagdasarjani* increased significantly. At 30 °C, these rates decreased, but not as much as they did at 20 °C, and there was a higher total consumption of prey at 25 °C.

The rate of attack and the handling time, both influenced by temperature changes and gauged by the predator’s effectiveness, are the two primary variables that alter the functional response [36,47]. The functional response parameter estimates of *A. swirskii* changed with temperature changes. The handling time/prey items at 26 °C were the smallest, while those at 14 °C were the greatest. The maximum searching efficiency (*a′*) was recorded at 26 °C, with the lowest search rate at 14 °C. According to Park et al. [48], at 25 °C, all species of phytoseiids had a stronger potential for predation on *T. urticae* eggs or larvae. Furthermore, *Neoseiulus californicus, N. longispinosus,* and *N. womersleyi* were evaluated using *T. urticae* eggs as prey at 30 and 35 °C.

The minimum temperature threshold for the attack rate of *A. swirskii* was evaluated by regressing the attack rate against the temperature (*R*^2^ = 0.976) at 14 °C. This finding aligns with previous laboratory observations by Farazmand and Amir-Maafi [41], who reported that the minimum temperature threshold for the attack rate of *A. swirskii* was 15 °C. They also stated that the phytoseiid has non-attacking behaviors at low temperatures. However, more searching and attacking occurs at higher temperatures, which may also occur in other predator–prey relationships. On the other hand, another study [49], when tested at 25, 30, and 35 °C, showed that the longest handling time (*T_h_*) of *A. swirskii* fed on *T. urticae* eggs on strawberries under laboratory conditions was recorded at 25 °C. In contrast, the lowest handling time and an increased attack rate were observed at 35 °C. The phytoseiid mite’s functional response may be altered by the size of the experimental unit and the plant species [50].

The second type of functional response is most common in phytoseiids due to an increasing density of *T. urticae* [46]. Our results were consistent with the study of Xiao et al. [20], who experimented on green bean leaves and stated that *A. swirskii* exhibited the type II functional response when feeding on the *T. urticae* at 26 °C. In addition, Fathipour et al. [37] noticed that 11, 16, and 21-day-old *A. swirskii* females showed a type II functional response to *T. urticae* at 25 °C. Midthassel et al. [33] also demonstrated that *A. swirskii* showed a functional type II response at 25 °C when fed on *Suidasia medanensis*.

In conclusion, the present study demonstrates a relationship between *A. swirskii* and *T. urticae*. Based on the available data, *A. swirskii* may be crucial in controlling *T. urticae* populations. The functional response in this study suggests that *A. swirskii* would be more effective at all studied densities of *T. urticae* at temperatures of 26–30 °C. However, it is essential to consider the results cautiously because laboratory data cannot fully explain these interactions as they can in the field; these experiments were performed under simplified laboratory conditions using small plant yards. In field and greenhouse conditions, adult *A. swirskii* mites can spread from one plant to another and interact with prey groups and other predators, which may significantly influence their efficiency. Further studies should add to the understanding that *A. swirskii* is an effective biological control agent for *T. urticae* in field and greenhouse environments.

## Figures and Tables

**Table 1:** *Tetranychus urticae* immature stages consumed by *Amblyseius swirskii* throughout one day at various temperatures and 60 ± 5% relative humidity.

°C	14	18	22	26	30	34	ANOVA	
Density	Mean ± SD. No. of *T. urticae* Consumed						F	Sig.
5	4.20 ± 0.42 ^a^	4.60 ± 0.52 ^b^	4.80 ± 0.42 ^bc^	5.00 ± 0.00 ^cd^	4.90 ± 0.32 ^bc^	4.90 ± 0.32 ^bc^	6.32	<0.001
15	11.80 ± 0.42 ^a^	13.80 ± 0.79 ^b^	14.10 ± 0.88 ^bc^	14.80 ± 0.42 ^d^	13.70 ± 0.48 ^bc^	12.90 ± 0.88^f^	23.74	<0.001
25	21.20 ± 0.92 ^a^	22.30 ± 0.95 ^b^	24.50 ± 0.53 ^c^	24.70 ± 0.48 ^cd^	23.80 ± 0.79 ^ce^	22.70 ± 0.95 ^bf^	29.73	<0.001
35	23.60 ± 0.52 ^a^	28.60 ± 0.84 ^b^	30.20 ± 0.79 ^c^	34.40 ± 0.52 ^d^	33.60 ± 0.70 ^e^	32.50 ± 0.85 ^f^	312.41	<0.001
45	24.30 ± 0.82 ^a^	30.70 ± 0.95 ^b^	31.50 ± 0.85 ^c^	41.20 ± 0.79 ^d^	40.50 ± 0.71 ^ed^	38.60 ± 0.84 ^f^	653.73	<0.001
55	27.40 ± 0.70 ^a^	32.10 ± 0.57 ^b^	33.70 ± 0.82 ^c^	43.80 ± 0.63 ^d^	42.20 ± 0.79 ^e^	41.30 ± 0.82 ^f^	822.06	<0.001
65	26.90 ± 0.99 ^a^	31.60 ± 0.70 ^b^	32.30 ± 0.48 ^bc^	42.70 ± 0.82 ^d^	41.60 ± 0.97 ^e^	40.20 ± 0.79 ^f^	637.86	<0.001
75	26.20 ± 0.92 ^a^	30.50 ± 0.71 ^b^	31.40 ± 0.70 ^c^	41.80 ± 0.92 ^d^	38.60 ± 0.70 ^e^	36.70 ± 0.82 ^f^	527.20	<0.001

According to the one-way ANOVA and LSD post hoc test, the means in rows followed by different letters are significantly different (*p* ≤ 0.05).

**Table 2:** Functional response parameter estimates for *A. swirskii* fed on *T. urticae* immature stages at diverse densities and different temperatures in laboratory settings, based on Holling’s type II model’s linearization.

°C	$T_{h}$	${T/T}_{h}$	$T_{s}$	$a^{\prime }$	$R^{2}$	Type
14	0.015	66.667	0.690	0.751	0.976	II
18	0.011	92.593	0.738	0.823	0.996	II
22	0.010	96.154	0.737	0.859	0.990	II
26	0.005	200.000	0.845	0.919	0.993	II
30	0.006	166.667	0.821	0.910	0.989	II
34	0.007	135.135	0.787	0.912	0.986	II

$T_{h}$ = handling time/prey item. T = total time spent. $T_{s}$ = total search time for all prey. $a^{\prime }$ = attack rate or searching efficiency. $R^{2}$ = quadratic coefficient.

## Data Availability

All data generated or analyzed during the study are included in this article. Further enquiries can be directed to the corresponding author.
